# Real Microgravity Influences the Cytoskeleton and Focal Adhesions in Human Breast Cancer Cells

**DOI:** 10.3390/ijms20133156

**Published:** 2019-06-28

**Authors:** Mohamed Zakaria Nassef, Sascha Kopp, Markus Wehland, Daniela Melnik, Jayashree Sahana, Marcus Krüger, Thomas J. Corydon, Hergen Oltmann, Burkhard Schmitz, Andreas Schütte, Thomas J. Bauer, Manfred Infanger, Daniela Grimm

**Affiliations:** 1 Clinic for Plastic, Aesthetic and Hand Surgery, Otto von Guericke University Magdeburg, D-39120 Magdeburg, Germany; 2 Department of Biomedicine, Aarhus University, DK-8000 Aarhus C, Denmark; 3 Department of Ophthalmology, Aarhus University Hospital, DK-8000 Aarhus C, Denmark; 4 Airbus Defence and Space GmbH, Airbus-Allee 1, D-28199 Bremen, Germany; 5 Gravitational Biology and Translational Regenerative Medicine, Faculty of Medicine and Mechanical Engineering, Otto von Guericke University Magdeburg, D-39120 Magdeburg, Germany

**Keywords:** microgravity, breast cancer cells, cytoskeleton, tubulin, F-actin, focal adhesion, E-cadherin, vinculin, live-cell imaging

## Abstract

With the increasing number of spaceflights, it is crucial to understand the changes occurring in human cells exposed to real microgravity (r-µ*g*) conditions. We tested the effect of r-µ*g* on MCF-7 breast cancer cells with the objective to investigate cytoskeletal alterations and early changes in the gene expression of factors belonging to the cytoskeleton, extracellular matrix, focal adhesion, and cytokines. In the Technische Experimente unter Schwerelosigkeit (TEXUS) 54 rocket mission, we had the opportunity to conduct our experiment during 6 min of r-µ*g* and focused on cytoskeletal alterations of MCF-7 breast cancer cells expressing the Lifeact-GFP marker protein for the visualization of F-actin as well as the mCherry-tubulin fusion protein using the Fluorescence Microscopy Analysis System (FLUMIAS) for fast live-cell imaging under r-µ*g*. Moreover, in a second mission we investigated changes in RNA transcription and morphology in breast cancer cells exposed to parabolic flight (PF) maneuvers (31st Deutsches Zentrum für Luft- und Raumfahrt (DLR) PF campaign). The MCF-7 cells showed a rearrangement of the F-actin and tubulin with holes, accumulations in the tubulin network, and the appearance of filopodia- and lamellipodia-like structures in the F-actin cytoskeleton shortly after the beginning of the r-µ*g* period. PF maneuvers induced an early up-regulation of *KRT8, RDX, TIMP1, CXCL8* mRNAs, and a down-regulation of *VCL* after the first parabola. E-cadherin protein was significantly reduced and is involved in cell adhesion processes, and plays a significant role in tumorigenesis. Changes in the E-cadherin protein synthesis can lead to tumor progression. Pathway analyses indicate that VCL protein has an activating effect on *CDH1*. In conclusion, live-cell imaging visualized similar changes as those occurring in thyroid cancer cells in r-µ*g*. This result indicates the presence of a common mechanism of gravity perception and sensation.

## 1. Introduction

Cancer is a burden of mankind with a high morbidity and mortality and is responsible for an estimated 9.6 million deaths, according to the World Health Organization (WHO) Global Cancer Observatory (GLOBOCAN) data indicated in 2018 [1]. The GLOBOCAN statistics from 2018 published that breast cancer is the most common cancer in women and the second leading cause of cancer deaths worldwide [1]. More women are diagnosed with breast cancer in 2019 than with any other cancer, besides skin cancer. In 2019, an estimated 268,600 women in the United States will be diagnosed with invasive breast cancer, and 62,930 women with in situ breast cancer [2]. In addition, an estimated 2670 men in the United States will be diagnosed with breast cancer.

Due to the high mortality, new ways must be taken to find new approaches and therapeutic strategies in cancer research. Experiments performed in the space environment are not typically the main topic of cancer researchers [3]. However, a stay in orbit on the International Space Station (ISS) provides physical conditions that are not achievable on Earth. Studying the mechanisms of microgravity-dependent cellular and molecular changes is necessary to improve space medicine and to develop new treatment strategies for cancer patients [4].

Gravity is the most familiar force in human life [5]. In space, the gravity force is lowered, resulting in microgravity (µ*g*). Although human cells do not have a gravity sensor, they can still sense µ*g* through the cytoskeleton [6]. The response of cells to early µ*g*, together with changes in actin and microtubules, has been reported for different kinds of cells [7,8,9,10]. Moreover, human thyroid cancer cells have demonstrated a response to early µ*g* by alterations in the cytoskeleton such as disturbance of F-Actin bundles, formation of lamellipodia- and filopodia-like structures and cellular detachment [11]. When cells are subjected to µ*g* for a longer duration, they tend to form three-dimensional (3D) aggregates, so-called multicellular spheroids (MCS) [12].

There are several options to study cells real microgravity (r-µ*g*), such as parabolic flights, sounding rockets missions, unmanned BION and photon spaceflights or a spaceflight in orbit or to the ISS with a commercial carrier. The Technische Experimente unter Schwerelosigkeit (TEXUS, TX) sounding rocket program, offered by the Deutsches Zentrum für Luft- und Raumfahrt (DLR, German Aerospace Center), offers researchers six minutes of µ*g* to conduct their experiments [11,13,14]. We participated in the TX54 mission with the main objective of study cytoskeletal alterations of breast cancer cells in r-µ*g*. Moreover, we attended the 31st DLR parabolic flight campaign (PFC) and investigated morphological changes and focused on the gene expression of selected genes of interest. The Michigan cancer foundation (MCF)-7 cell line was chosen as a suitable candidate for both the TX 54 mission and the PFC. As an alternative to conducting experiment in r-µ*g*, which often is very expensive and time consuming, researchers may choose to investigate biological processes in simulated µ*g*. The simulation of µ*g* is achievable by using the rotating wall vessel (RWV), the random positioning machine (RPM), a 2D or 3D clinostat, and magnetic levitation [15]. These special conditions had been applied to study changes in cell growth and the function of different benign cell types and cancer cells, and may to some extent resemble the findings provided by r-µ*g* [16]. 

The MCF-7 cell line had been investigated for several times under altered µ*g* conditions in space and on Earth. The MCF-7 cell line showed a robust behavior in µ*g*, and MCF-7 cells had been tested in space in a Photon capsule [17]. The MCF-7 cells reacted by exhibiting alterations in microtubules [17]. In addition, the MCF-7 cells exerted a loosening of the perinuclear cytokeratin network [18]. RPM-exposure experiments of the MCF-7 cells revealed changes in their growth behavior.

In this paper, we report for the first-time live-cell imaging of breast cancer cells on board a sounding rocket (TX 54). Live-cell imaging was performed with a spinning-disc Fluorescence Microscopy Analysis System (FLUMIAS) [11]. The objective of this study was first to focus on changes in the microtubule and F-actin cytoskeleton. Second, we investigated the changes in expression, distribution and localization of selected proteins by indirect immunofluorescence of fixed cells during µ*g*. Third, we examined MCF-7 cells exposed to PF maneuvers and addressed changes in the gene expression patterns of genes encoding for components of the cytoskeleton, the extracellular matrix (ECM), focal adhesion molecules and cytokines.

## 2. Results

The TEXUS program is an established DLR-sponsored sounding rocket program which offers researchers precious µ*g* time to test their hypotheses [19]. The sounding rocket has the advantage of providing a relatively longer time (6 min) period of r-µ*g* compared to the parabolic flights. Moreover, it has only one period of hypergravity (hyper-*g*) and vibration at the beginning prior to the r-µ*g* phase.

### 2.1. TEXUS 54 Sounding Rocket Mission: “Live-Cell Imaging of Human Breast Cancer Cells in Short-Term Weightlessness”

The cytoskeleton is a highly dynamic structure playing a crucial role in adaptation and cell signaling processes in µ*g*. We first examined human MCF-7 breast cancer cells on a sounding rocket and studied the early cytoskeletal alterations (F-actin, α-tubulin). 

**Figure 1:**
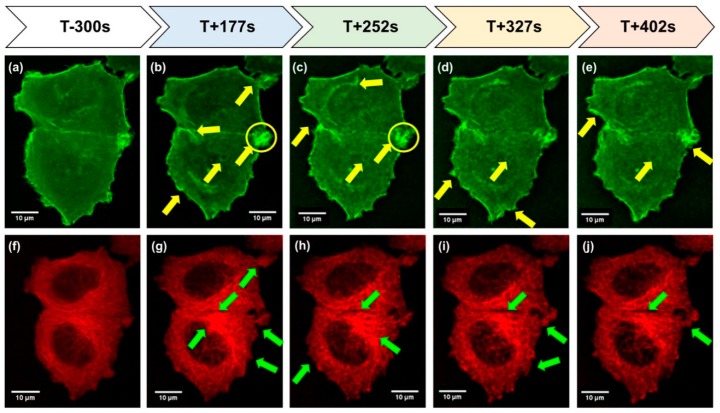
Time course and images of FLUMIAS on TEXUS 54 (40×/1.2). The MCF-7 breast cancer cells 5 min before launch (T-300 s) of the rocket and during the r-μ*g* phase (T + 177s–T + 402s). The yellow arrows show the changes in F-actin (**a**–**e**; green fluorescence). The yellow circles include an area with F-actin accumulations. Filopodia and lamellipodia are found after 150s, which are more pronounced with time. The green arrows indicate changes in α-tubulin (**f**–**j**; red fluorescence). The tubulin network reveals holes after 150s and a looser structure.

MCF-7 cells exhibited three forms of growth when cultured for 24 days and five days under s-µ*g* conditions. One part grew adherently on the cell culture flask bottom, a second group formed duct-like multicellular spheroids and a third group revealed compact spheroids on the RPM after a five-day exposure, whereas after 24h only adherent cells and compact MCS were visible [20,21]. The MCF-7 cells were transfected with a Sleeping Beauty transposon-based (pSB-LAGICT) expression construct to visualize F-actin and α-tubulin. The LAGICT (LifeAct-eGFP-IRES-mCherry-Tubulin) expression cassette enables simultaneous examination of F-actin and α-tubulin, through co-expression of Lifeact GFP and mCherry-tubulin fusion proteins, respectively. Transfected MCF-7 cells were examined with the FLUMIAS microscope with 488 nm and 568 nm diode lasers prior to launch and during r-µ*g*. All the images taken at r-µ*g* were compared to control images (Figure 1) which were taken before launch. We showed that MCF-7 cells respond to r-µ*g* within four minutes and demonstrate similar changes as the FTC-133 thyroid cancer cells studied in previous campaigns [11]. This indicates a general gravitational mechanism in human cancer cells. During the r-µ*g* phase of the TEXUS flight, various changes in the cytoskeleton were seen, including a clear influence on the F-actin bundles and the appearance of filopodia/lamellipodia-like structures (Figure 1).

### 2.2. Immunostaining of MCF 7 Cells Exposed to r-µg during the TEXUS 54 Sounding Rocket Mission and Fixed in Orbit 

In addition to the live-cell imaging studies of the transfected MCF-7 cells, regular MCF-7 cells were seeded into 18-well Ibidi slides which were fixed with 4% PFA at the end of the *µg* period and the hyper-g period. These slides were compared to a control slide fixed with 4% PFA on ground. Thus, we had the opportunity to investigate the changes in expression and distribution of the designated proteins. We tested the antibodies MMP9, VEGFA (c-term), IL-6 and IL-8. Phalloidin rhodamine and DAPI stains were used additionally for all the slides from the TX 54 mission. 

**Figure 2:**
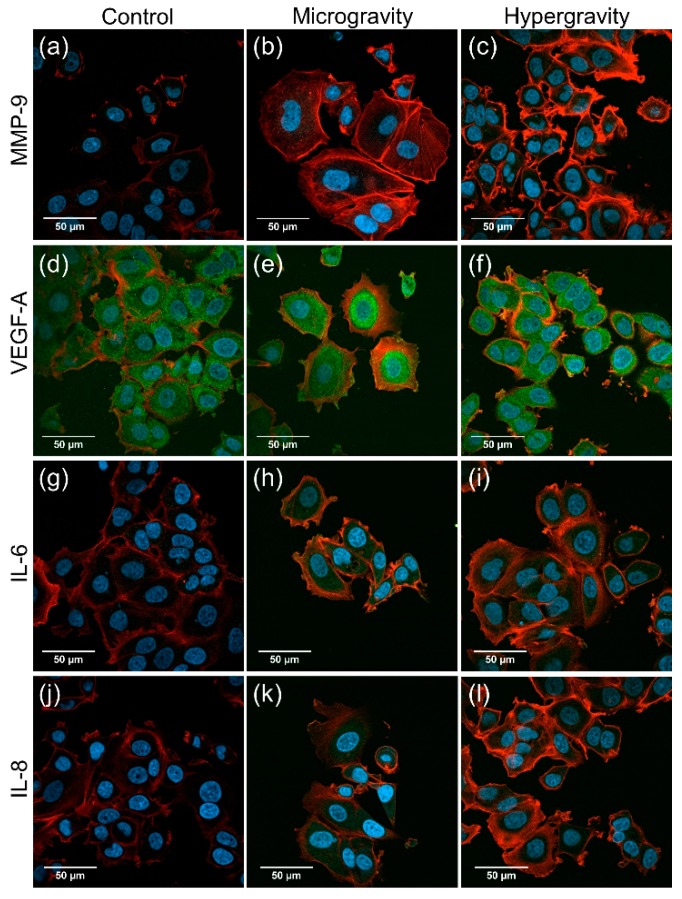

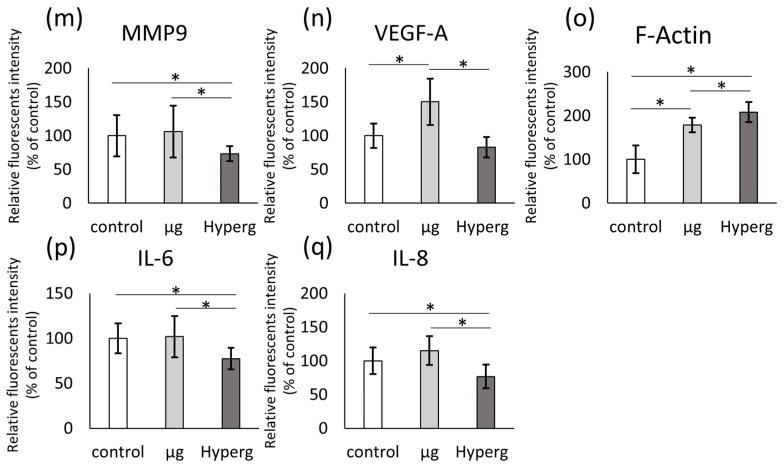
Indirect immunofluorescence staining of PFA-fixed cells from the TEXUS 54 sounding rocket mission. 2500 MCF-7 cells were seeded into each well of the 18 well Ibidi slides. The cells were fixed either at the end of the hyper-*g* period or at the end of the µ*g* period. The two conditions were compared to 1*g*-ground control samples. The confocal laser scanning microscopy images (**a**–**l**) show either MMP9, VEGF-A, IL-6 or IL-8 in green, DAPI (in blue) and F-Actin (in red). Scale bar is equal to 50 µm. The intensity of the staining was quantified by ImageJ (**m**–**q**). All data are shown as mean ± SD, *n* = 10–14, with significance indicated by * *p* < 0.05.

Upon visual inspection of the microscopic images, there was no apparent difference in the protein distribution between the different conditions for all the tested antibodies (Figure 2a–l). In order to provide an indication on whether the level of the visualized proteins may have changed, the microscopic images were analyzed at the end of the hyper-*g* and µ*g* phases in comparison to the controls by using the ImageJ program. At the end of the hyper-*g* phase, the analysis indicated that the level of F-actin was increased (Figure 2c,f,i,l,o) compared to the controls (Figure 2a,d,g,j), while the level of MMP9 (Figure 2a,c), IL-6 (Figure 2g,i) and IL-8 (Figure 2j,l) apparently was reduced (Figure 2m,p,q). VEGF exhibited no changes at the end of hyper-*g* phase (Figure 2d,f,n). Furthermore, the microscopic images at the end of the r-µ*g* phase were also investigated in comparison to the controls. The levels of VEGF and F-actin appeared to be increased (Figure 2n,o), while the levels of MMP9, IL-6 and IL-8 apparently were unchanged (Figure 2m,p,q).

### 2.3. Results of the 31st DLR Parabolic Flight Campaign: “Effects Of Short-Term Microgravity on Human Breast Cancer Cells”

The PFCs were organized by the DLR in close collaboration with the company Novespace, Bordeaux-Mérignac, France. A parabolic flight consisted of 31 consecutive parabolas. Each parabola contains two phases of 1.8 *g* hyper-*g* spanning one phase of 10^−2^ *g* r-µ*g*. The first phase of a parabola begins with flying at 45 degrees for 22 s known as pull-up, followed by decreasing thrust and following the trajectory of a parabola for 22 s of free-fall. Thus, the r-µ*g* phase lasts for the 22 s of the free-fall. The last phase is known as pull-out which begins pulling out the plane to fly back on a horizontal line trajectory. The pull-up phase lasts for 22 s with hyper-*g* of 1.8 *g* [16,22,23]. Not only does the parabolic flight have the advantage of offering valuable r-µ*g* time to researchers, but also it gives the scientists access to their experiment on board of the flight.

**Figure 3:**
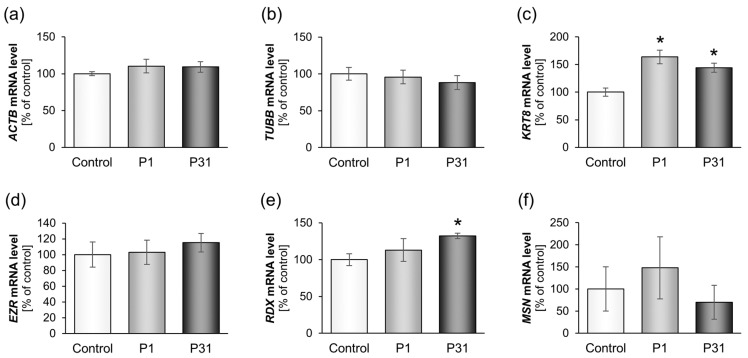
Influence of short-term microgravity on the gene expression (mRNA) of cytoskeletal factors. The data are given as mean ± standard deviation. * *p* < 0.05 vs. Control.

**Figure 4:**
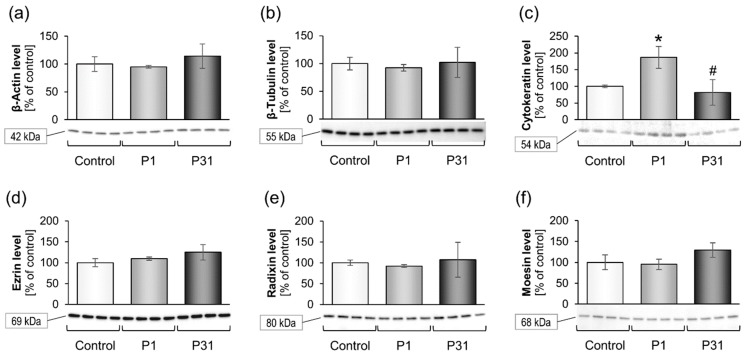
Influence of short-term microgravity on the protein accumulation of cytoskeletal factors. The data are given as mean ± standard deviation. * *p* < 0.05 vs. Control; ^#^ *p* < 0.05 vs. P1.

**Figure 5:**
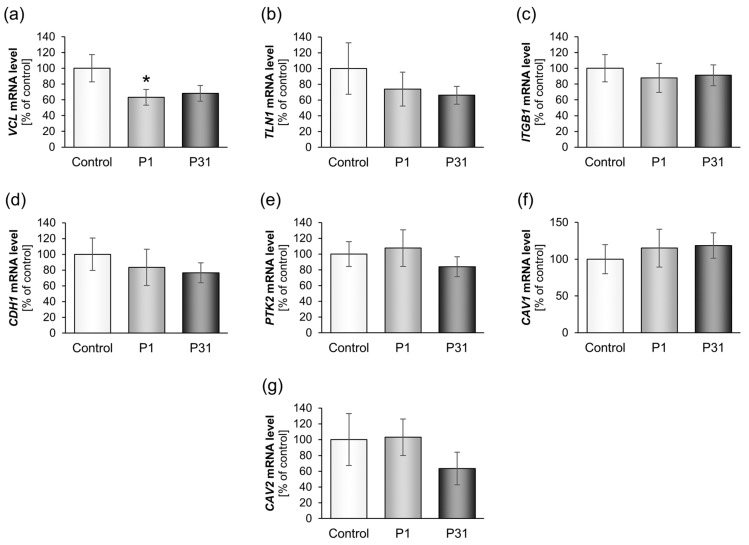
Influence of short-term microgravity on the expression (mRNA) of focal adhesion complex components. The data is given as mean ± standard deviation. * *p* < 0.05 vs. Control.

**Figure 6:**
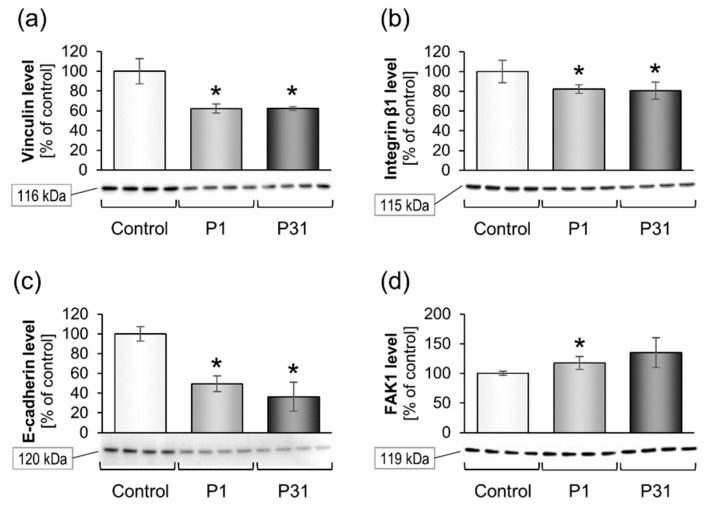
Influence of short-term microgravity on the protein accumulation of focal adhesion complex components. The data is given as mean ± standard deviation. * *p* < 0.05 vs. Control.

**Figure 7:**
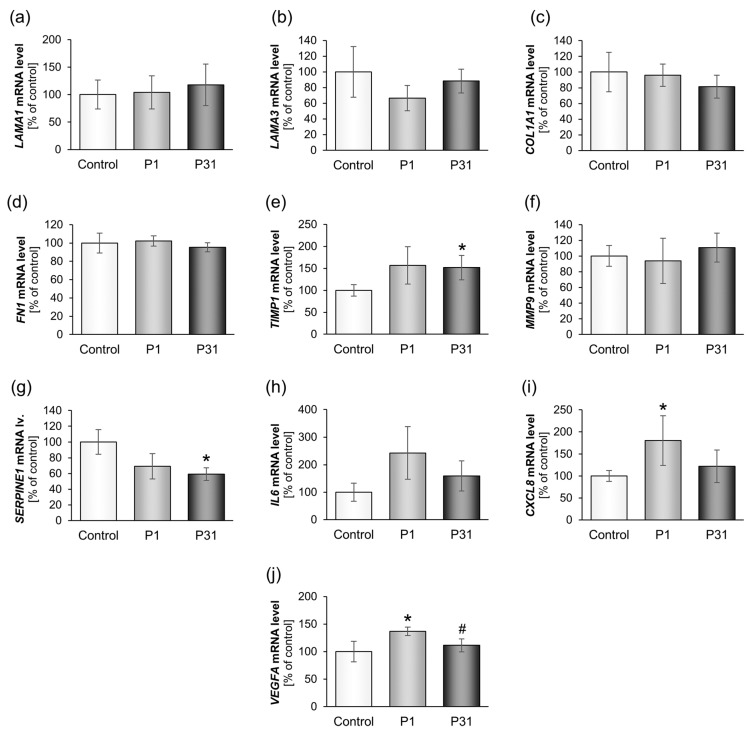
Influence of short-term microgravity on expression of different ECM and cytokine genes. * *p* < 0.05 vs. Control; ^#^ *p* < 0.05 vs. P1.

**Figure 8:**
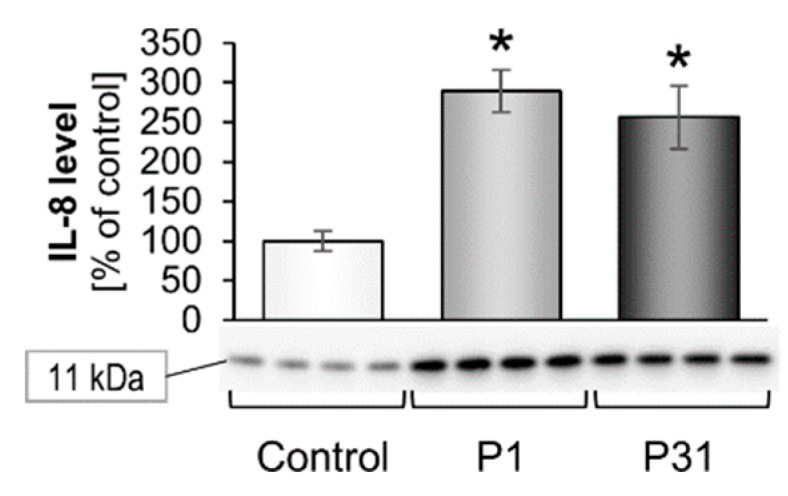
Influence of short-term microgravity on the protein accumulation of IL-8. The data is given as mean ± standard deviation. * *p* < 0.05 vs. Control.

**Figure 9:**
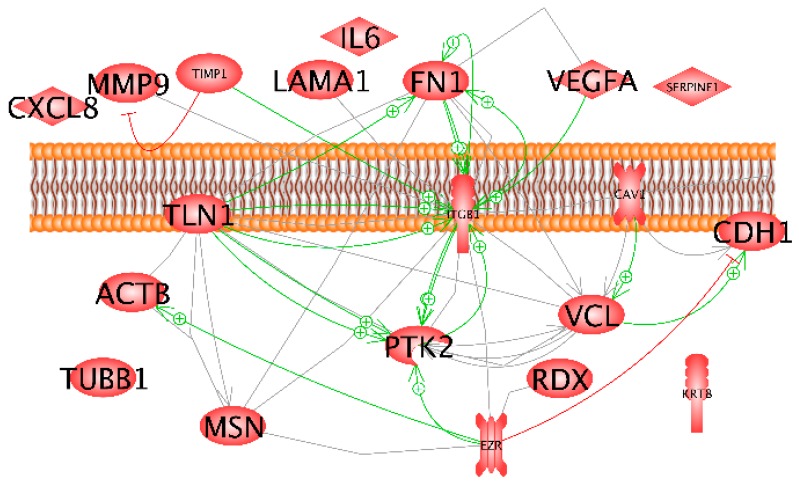
Interaction and localization of proteins detected in the cells by Western blot (red margins). Solid lines indicate the bond. Filled arrows show the directional interaction and dashed arrows show the influence. + Signs indicate activity − inducing effect and red lines indicate inhibition.

**Figure 10:**
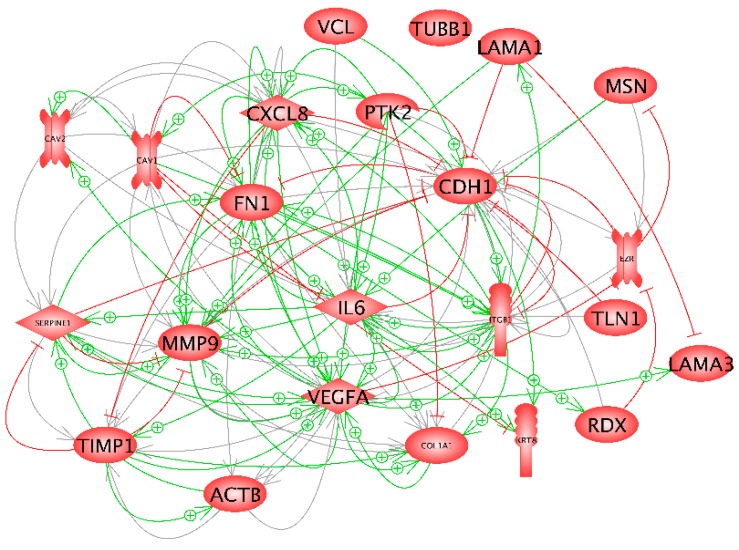
Mutual interactions of expression of the genes examined with qPCR and shown in Figure 2, Figure 3 and Figure 4. + Signs indicate an activity-enhancing effect and red lines indicate inhibition. The interaction networks were created using Elsevier Pathway Studio v11.

The 31st DLR PFC took place in March 2018 at the company Novespace at the Bordeaux-Mérignac airport in France. The breast cancer cells (MCF-7 cell line) were examined after parabola 1 (P1) and after 31 parabolas (P31). At the same time, the ground control experimentation took place in the laboratories of Novespace. Subsequently, the fixed cells were transported to the Otto von Guericke University Magdeburg, Germany, where they were examined by molecular biology. We performed qPCR, Western blot and pathway analyses. The results are shown in the following Figure 3, Figure 4, Figure 5, Figure 6, Figure 7, Figure 8, Figure 9 and Figure 10. Figure 3, Figure 4, Figure 5, Figure 6, Figure 7 and Figure 8 each show the following groups: static ground controls (1*g*), P1 and P31.

#### 2.3.1. Studies on Cytoskeletal Genes

Figure 3 gives the expression of the cytoskeletal genes and Figure 4 shows the levels of the corresponding proteins. There were no changes after P1 for the *ACTB*, *TUBB*, *EZR*, *RDX* and *MSN* mRNAs. However, there was a significant up-regulation of *KRT8* after P1 and P31 compared to 1*g* (Figure 3c) and of *RDX* after P31 (Figure 3e). The cytokeratin protein synthesis was also significantly increased after the first parabola but returned to control levels after 31 parabolas (Figure 4c).

#### 2.3.2. Altered Expression of Genes of the Focal Adhesion Complex

Furthermore, we examined the mRNA expression and protein content of focal adhesion molecules (Figure 5 and Figure 6). The *VCL* gene and corresponding vinculin protein were significantly reduced under r-µ*g* (Figure 5a and Figure 6a). *TLN1, ITGB1, CDH1, PTK2, CAV1* and *CAV2* remained unchanged under r-µ*g*, while the E-cadherin protein (Figure 6c) as well as β_1_-integrin protein (Figure 6b) were significantly reduced. FAK1 protein was elevated after P1 (Figure 6d).

#### 2.3.3. Changes of Extracellular Matrix and Cytokine Gene Expression

The results on the extracellular matrix proteins are shown in Figure 7 and Figure 8. There were no changes in the gene expression for *LAMA1, LAMA3, COL1A1, FN1, MMP9* and *PAI1* (*SERPINE1*). The *TIMP1* mRNA was significantly elevated after P31 compared with ground control samples (Figure 7e). In addition, we investigated the expression of the cytokines IL-6 and IL-8, which play a major role in spheroid formation. The *IL6* mRNA was not significantly changed, but there was a tendency of an up-regulation after P1 and P31 (Figure 7h). In contrast, *CXCL8* was significantly up-regulated after P1, and slightly elevated after P31 (Figure 7i). A similar behavior showed the corresponding IL-8 protein synthesis, which was significantly increased after P1 and P31 (Figure 8). Moreover, a significant elevation of the *VEGFA* mRNA was detectable (Figure 7j).

#### 2.3.4. Pathway Analyses

The results of the pathway analysis given in Figure 9 indicate a clear role for *ITGB1, FN1*, *TLN1, CDH1, PTK2* and *VCL* in gravity sensing of MCF-7 breast cancer cells exposed to short-term r-µ*g*.

*FN1, VEGFA, TLN1* and *TIMP1* proteins are positively influencing β_1_-integrin which is inducing *PTK2* and fibronectin. *TLN1* is interacting with *PTK2* and *FN1*. Vinculin is activating E-cadherin, whereas ezrin has a negative influence on *CDH1*. Moreover, *TIMP1* is inhibiting *MMP9*.

The interaction network shown in Figure 10 indicates a fast significant up-regulation of *KRT8, RDX, TIMP1* and *CXCL8*, while *VCL* is down-regulated. Simultaneously, *TLN1* and *CDH1* indicate a slight but non-significant reduction of gene expression. Considering the red lines together with the qPCR results cell adhesion via *CDH1* seems to be attenuated. Various genes demonstrate an inhibitory effect on *CDH1* (Figure 9).

## 3. Discussion

In this study we investigated MCF-7 breast cancer cells in real microgravity using two experimental platforms offered by a sounding rocket mission and a parabolic flight. Live-cell imaging visualized similar changes as those occurring in thyroid cancer cells, when they were exposed to r-*µg*. Our results now indicate the presence of a common cellular mechanism for sensing gravity. After entering microgravity, the MCF-7 cells showed a rearrangement of F-actin and tubulin comprising holes, accumulations in the tubulin network, and the appearance of filopodia- and lamellipodia-like structures. Up-regulation of *VEGF* and down-regulation of E-cadherin during parabolic flight maneuvers indicated a temporary change to a more invasive phenotype of MCF-7 cells. This finding also supports earlier data gained from thyroid cancer cells and may hint at a general cellular answer of cancer cells to short-term microgravity.

There is evidence that in vitro cell cultures respond to altered gravity conditions. Studies suggest that this response might be involved in the physiological changes and fundamental health problems of humans during spaceflight [24]. Microgravity alters adhesion, migration, proliferation, differentiation, growth, signaling and gene expression [25]. The cytoskeleton is discussed as the initial gravity sensor [26]. For example, bone cells exposed to r- and s-µ*g* show changes in their cytoskeleton and focal adhesions, two major mechanosensitive structures [27,28]. Similar results were obtained when investigating thyroid cancer and melanoma cells cultured under µ*g*-conditions [14,29].

Live-cell imaging of benign and tumor cells is possible using the FLUMIAS microscope which was flown on parabolic flight missions, sounding rocket missions and a more advanced version to the ISS [11,30].

### 3.1. Cytoskeletal Alterations Visualized during the TEXUS 54 Mission

The principal aim of our experiment onboard the TX 54 sounding rocket was to investigate the effect of r-µ*g* on the cytoskeleton of human breast cancer cells.

During the flight, MCF-7 cells exhibited alterations in the F-actin and microtubule cytoskeleton. The appearance of filopodia- and lamellipodia-like structures shortly after the beginning of the r-µ*g* period is comparable to the results which we have obtained with FTC-133 thyroid cancer cells during the TX 52 mission [11]. This data indicates that the gravi-sensing is similar in both cancer types. We could visualize impressing changes of alpha-tubulin during the r-µ*g* phase. This supports earlier results [20]. Investigating suspensions of purified tubulin, Moos et al. [31] found a significant difference between microtubules assembled in the 30 s µ*g* phase and the 2*g* hyper-*g* phase of a PF. These data reveal that microtubule polymerization is altered by altered gravity. These results were substantiated by sounding rocket [32] and shuttle flight [33] experiments.

In addition, an immunofluorescence staining on MCF-7 cells was performed. The cell fixation was done automatically during the r-µ*g* phase of the TX 54 mission. The quantitative analysis of VEGF exhibited an up-regulation of the protein in the r-µ*g* condition compared to the controls. We also measured an accumulation of F-actin. While VEGFA was up-regulated only in the r-µ*g* condition, F-actin was increased in both the hyper-*g* and r-µ*g* conditions. The significant up-regulation of a protein in both µ*g* and hyper-*g* conditions indicates that hyper-*g* was the main inducer for the up-regulation, because the hyper-*g* period precedes the µ*g* period. The F-actin content was elevated in A431 epidermoid carcinoma cells after a 7-min µ*g*-exposure during a sounding rocket flight [34]. Boonstra concluded that the actin microfilament system is sensitive to altered gravity conditions and that its remodeling may affect signal transduction [34]. Reflecting our results, we can support this hypothesis.

Therefore, we can conclude that only VEGF showed a clear up-regulation in r-µ*g* compared to the control. This data fits to earlier results obtained during a PF mission, where *VEGF* was significantly up-regulated after P1 and P31 [16]. VEGF is a key inducer of angiogenesis and enhances spreading and metastasis [35,36]. Serum VEGF was significantly elevated in patients with metastatic differentiated thyroid cancer but not in those with poorly differentiated thyroid cancer metastases [37]. The release of VEGF by FTC-133 exposed to space conditions during the Shenzhou-8 spaceflight was high but not significantly different in 1*g* samples and r-µ*g* samples, but the gene expression of *VEGFA* was down-regulated after a 10-day mission [16].

### 3.2. MCF-7 Breast Cancer Cells Exposed to PF Maneuvers during the 31st DLR PFC

Earlier experiments investigating chondrocytes, endothelial cells, and thyroid cancer cells revealed that a short-term r-µ*g*-exposure (22 s) during PF maneuvers induced early cytoskeletal changes and an altered gene expression pattern in these different cell types [22,38,39]. These studies showed that the gravi-response of cancer and benign cells occurred very early, within the first few seconds [22,38,39]. Several gravi-sensitive signaling elements, such as AMP-activated protein kinase alpha 1 and integrins are involved in the reaction of endothelial cells to altered gravity conditions [38]. Down-regulated *MTSS1* and up-regulated *LIMA1* were key factors stabilizing the cytoskeleton of tumor cells under µ*g* conditions [39].

ECM proteins, focal adhesion and cytoskeletal components form a dynamic network interacting with signaling molecules as an adaptive response to altered gravity. These focal adhesions are integrin-containing structures that form mechanical links between intracellular actin bundles and the extracellular space of a cell. Focal adhesions are dynamic protein complexes through which the cytoskeleton of a cell connects to the ECM. The adhesion dynamics play a central role in cellular migration. Focal complexes are formed at the leading edge of the cell in lamellipodia. 

We had first investigated the gene expression of cytoskeletal factors. Most cytoskeletal genes were not significantly altered but there was a significant up-regulation of the *KRT8* mRNA after P1 and P31. This finding is similar to earlier results with thyroid cancer cells [39]. 

Studying the genes of the focal adhesion complex we found a decrease in E-cadherin protein as well as β_1_-integrin protein. Changes in the focal adhesion complex were also found in cells exposed to s-µ*g* and r-µ*g*. Tan et al. [29] reported about a reduction in focal adhesions in melanoma cells exposed to s-µ*g*. In space on board the SJ-10 satellite, the mechanosensitive molecules β_1_-integrin, β-actin, α-tubulin, and others were elevated, whereas among others vinculin was down-regulated in bone marrow-derived mesenchymal stem cells [40]. In addition, the authors observed an accumulation of microtubules and vimentin through the altered expression and location of focal adhesion complexes [40]. 

Several ECM genes such as *FN1* were not altered during short-term µ*g* in MCF-7 breast cancer cells. This is different to the results which we obtained from low-differentiated ML-1 thyroid cancer cells [39]. During this PFC we detected an up-regulation of the *FN1* mRNA, which indicates a cancer type-specific reaction to µ*g*.

β_1_-integrin, fibronectin, and PTK2 play essential roles, when cells bind to the extracellular matrix. β_1_-integrin is involved in the proliferation and differentiation as well as in the development of epithelial tissues [41]. It is important for adhesion dynamics and essential for the control of cell migration and, therefore, is a marker for a poor prognosis in breast cancer [41]. β_1_-integrin forms heterodimers with various α-types integrins. Some of these dimers are able to bind to fibronectin. After binding, signals are forwarded to FAK1 (Focal adhesion kinase 1 coded by *PTK2*) and talin (TLN1) [42].

Binding of β_1_-integrin to fibrillar fibronectin promotes the phosphorylation of STAT3, which is known to participate in the induction of the epithelial mesenchymal transition [43]. In addition, β_1_-integrin facilitates the secretion of fibronectin in a way which is poorly understood. Fibronectin remaining intracellular is directly interacting with the actin cytoskeleton [43], which is changed when the cells are exposed to microgravity [20]. Moreover, β_1_-integrin controls the VE-cadherin localization and blood vessel stability in the mouse retina [44]. VEGF can promote angiogenesis through up-regulation and/or activation of integrins [45]. The VEGF activity is dependent on β_1_-integrin function. 

FAK1 and talin are members of the focal adhesion complexes. Talin mediates cell-cell adhesion linking the integrins to the actin cytoskeleton and in the activation of integrins [46]. Talin was differentially regulated in AD and MCS of thyroid cancer cells exposed to simulated microgravity [47]. FAK1 is a cytoplasmic protein tyrosine, kinase which is found to be concentrated in the focal adhesions. It promotes the organization of the fibronectin matrix and fibrillar adhesions [48]. In addition, FAK1 triggers the recruitment of talin to nascent adhesions independently of integrin. It plays a key role in the migration process and FAK1 inhibition decreases mobility and metastasis. The β1-Integrin-FAK pathway is involved in cell survival and on the role of FAK in breast cancer development and progression [49]. In this study, the *PTK2* mRNA was stable in the breast cancer cells, but the corresponding protein showed an elevation after P1 (Figure 6d). This is different to the data obtained with thyroid cancer cells, where the *PTK2* mRNA in FTC-133 thyroid cancer cells was differentially expressed in the TEXUS samples, but the FAK1 protein content was significantly reduced in RPM samples [14]. 

The pathway analysis of the investigated genes revealed important roles of *CDH1* and *VEGFA* in the network. These genes are under the influence of IL-6 and IL-8, which both exert inhibitory effects on CDH1 and promoting effects on VEGFA. IL-6 promotes e-cadherin repression [50] and treating human gastric carcinoma cells with exogenous IL-8 decreased expression of E-cadherin mRNA [51]. IL-6 and IL-8 induce the expression of VEGF [52,53]. The *CDH1* gene encodes e-cadherin, which establishes cell-cell junctions [54]. After a 2-week RPM-exposure, E-cadherin, was diminished in MCF-7 MCS cells, where proteins of the E-cadherin autodegradation pathway were enhanced as well as c-Src (proto-oncogene tyrosine-protein kinase c-Src) [55]. Blocking the E-cadherin activity by specific antibodies promoted spheroid formation. As indicated by the high number of red lines shown in Figure 10, *CDH1* gene expression is strongly controlled by a number of proteins. Of the investigated components, only vinculin has a positive modulatory influence (green arrow) [56].

But, in addition to IL-6 and IL-8, EZR, talin, PTK2, ITGB1 and MMP9 were already found to have inhibitory effects. E-cadherin and EZR are involved in tumor growth. In a patient study, the ezrin expression was upregulated, while that of E-cadherin was decreased in breast cancer as compared to the control specimen. Ezrin expression was negatively correlated to the E-cadherin expression in a subpopulation of breast cancer patients with a high expression of ezrin [ezrin(high)] and a low expression of E-cadherin [E-cad(low)] [57]. Talin regulates the stability of E-cadherin transcriptional repressors [58]. An increased expression of E-cadherin was observed after reducing the level of β_1_-integrin in B16-F10 melanoma cells [59]. Treatment with recombinant MMP-9 or transient expression of MMP-9 is sufficient to reduce E-cadherin levels in differentiated ovarian tumor cells [60].

*VEGFA* encodes the vascular endothelial growth factor, which supports (neo)-vascularization of healthy and malignant tissues [61]. As mentioned above, IL-6 and IL-8 favor its expression. In addition, human astrocytoma cells expressed increased levels of fibronectin and VEGF upon transformation with a versican G3 construct [62] and VEGF expression was positively linked to MMP-9 levels in gastric carcinoma [63]. Overexpression of TIMP1 and PAI-1 in endothelial cells blocks vascular tube regression [64].

### 3.3. Microgravity-Induced Cytoskeleton Changes and Potent Physiological Responses

**Figure 11:**
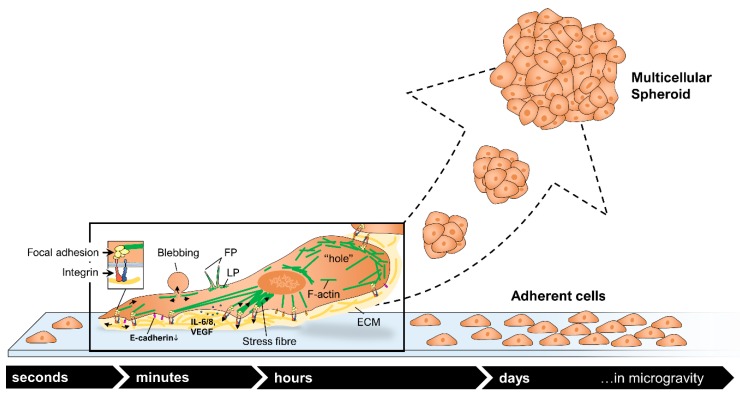
Alterations of adherently growing cancer cells after exposure to microgravity (modified from [65]). The figure in the box shows changes at the microscopic level. F-actin is displayed as green lines, the ECM in yellow. FP: filopodia, LP: lamellipodia.

The cytoskeleton directs a number of essential cell functions, such as maintenance of cell shape, support of vacuole formation or fixation of organelles. The cytoskeleton is dynamic and not static, and it can disassemble and reassemble to contribute to cell mobility (Figure 11). It is involved in migration which is necessary for tissue repair and tumor spreading, progression and metastasis [65]. It is important for cellular signaling.

In this study, we demonstrate for the first time using live-cell imaging in real µ*g* that MCF-7 cells reveal a rearrangement of the F-actin and microtubule cytoskeleton with holes, accumulations in the tubulin network, and the appearance of filopodia- and lamellipodia-like structures in the F-actin cytoskeleton (Figure 1). In lamellipodia are ribs of actin called microspikes spreading beyond the lamellipodium frontier are then called filopodia, which are involved in cell adhesion and migration, healing processes and others. The initiation and elongation of filopodia is depending on polymerization, convergence and crosslinking of actin filaments [66]. Filopodia and filopodia-like structures are involved in 3D cell migration and thus in tumor dissemination, growth and metastasis [67]. Filopodia are normally situated at the front of invading cancer cells [68,69] and filopodia-like structures promote cancer cell survival at metastatic sites [70,71]. In Figure 11 the possible mechanism is demonstrated how these µ*g*-induced alterations of the cytoskeleton are involved in 3D growth and in the formation of multicellular spheroids, which resemble in vivo microtumors and metastases [65].

In this study PF maneuvers induced a downregulation of *VCL* mRNA and the corresponding protein in MCF-7 breast cancer cells. Pathway analyses indicated that VCL protein has an activating effect on *CDH1*. E-cadherin is involved in cell adhesion and in tumorigenesis. Changes in the E-cadherin protein synthesis can lead to tumor progression. In addition, blockage of E-cadherin and a down-regulation of *CDH1* leads to enhanced spheroid formation of MCF-7 breast cancer cells [55,65]. 

r-µ*g*-induced changes in the cytoskeleton and/or in focal adhesion components of human cells as for example cancer cells and normal cells like osteoblasts, chondrocytes and cancer cells are the two major mechanosensitive responses [8,14,28]. The cytoskeleton responds to changes in the mechanical environment because of its connection to the ECM through focal adhesions. Exposure of human cells to *µg* impaired their cytoskeleton stability and reduced cellular tension, as well as focal adhesion formation and stability [8].

The ECM-membrane receptors-cytoskeleton system is known to be involved in cancer and metastasis [72]. Growth factor receptors, adhesion proteins and ion channels in association with the sub-membranous system of the actin cytoskeleton control the actin microfilament’s force generating capacity. Perturbations of the actin microfilament system in cell motility and migration and their role in cancer pathophysiology are currently a hot topic [72].

The exact mechanisms how the cells transform the mechanical signal (microgravity) into a biochemical signal is still not known. The widely acknowledged tensegrity model hypothesis proposed by Ingber [73] explains that the cells are hardwired by the cytoskeleton components. The cells are spanned open and are under continuous tension [26,73]. An imbalance between adhesion and the cytoskeleton network might induce a cell shape change and has a direct impact on cell signaling [26,73]. This theory is supported by data of cytoskeletal changes in various benign and malignant cell types after short-term exposure to microgravity [11,16,21,22,38,74].

When microgravity conditions influence human adherent MCF-7 breast cancer cells, the microtubules (tubulin) rapidly reorient themselves and actin stress fibers increase in density in order to reinforce their mechanical strength. These changes in the F-actin and in microtubule network are followed by changes in adhesion, shedding of membrane receptors, cell detachment, migration, growth behavior, differentiation, and apoptosis [12,20,38,75]. These physiological changes have been detected in real microgravity and on Earth using various µ*g*-simulation devices [15].

In summary, an early up-regulation of *KRT8, RDX, TIMP1, CXCL8*, and down-regulation of *VCL* were found after the PF maneuvers. E-cadherin protein was reduced after the PF maneuvers. This result is very important because E-cadherin is not only involved in the cell adhesion process but plays a significant role in tumorigenesis. Changes in the E-cadherin protein can lead to tumor progression. VCL protein has an activating effect on *CDH1* (Figure 9). However, many of the genes investigated lead to an inhibition of *CDH1* (Figure 10). *CXCL8* is also important for tumor progression and the early up-regulation of the cytokine supports this thesis.

## 4. Materials and Methods 

### 4.1. Cell Culture

The MCF-7 breast cancer cells were purchased from the American Type Culture Collection (MCF7, ATCC^®^ HTB-22™). They were cultured in RPMI 1640 (Life Technologies, Paisley, UK), 10 % FCS (Sigma Aldrich, Steinheim, Germany) and 1% penicillin/ streptomycin (Life Technologies, New York, NY, USA). The Lifeact-eGFP-IRES-mCherry-Tubulin MCF-7 expressing cells were cultured in the same medium, in addition to G418 (Geneticin) to allow the growth of the stably transfected cells. The cells were cultured in T-75 flasks and the cells were harvested and seeded into other flasks every 3-5 days to prevent confluence.

### 4.2. Construction of An Expression Cassette to Visualize F-actin and α-tubulin

We constructed a pcDNA3.1 LifeAct-eGFP-IRES-mCherry-Tubulin (pLAGICT) expression cassette for visualization of F-acting and α-tubulin. In order to construct this expression cassette, pUC57 plasmid was ordered from GenScript. The required cassette LifeAct-eGFP-IRES-mCherry-Tubulin was excised from the pUC57 plasmid with NotI/XbaI restriction enzymes. The excised part was further purified by gel extraction and ligated into pcDNA3.1 (+) (Invitrogen, Carlsberg, CA, USA). The constructed plasmid was transformed into ultra-competent *Escherichia coli* cells to produce more copies of the plasmid. QIAprep spin column (Qiagen, Hilden, Germany) was used to extract the plasmid from the bacteria. More details about the construction of the expression cassette are mentioned in [11,76].

### 4.3. Generation of MCF 7 Cells Expressing Lifeact-eGFP-IRES-mCherry-Tubulin

The MCF-7 cell line was stably transfected using a Sleeping Beauty (SB) transposon-based vector containing the LAGICT expression cassette for the visualization of F-actin and α-tubulin as described in [11,77,78]. In brief, LifeAct-eGFP-IRES-mCherry-Tubulin was excised from the pLAGICT plasmid with NotI/XbaI restriction enzymes, and sub-cloned into a Sleeping Beauty transposon-based vector pT2/CMV-linker-SV40-Neo [78,79] containing a linker enabling insertion of the NotI-LAGICT-Xba1 fragment excised from the pLAGICT vector. The resulting plasmid, pT2/CMV-LAGICT-SV40-Neo, was entitled pSB-LAGICT. In order to provide stable expression of the LifeAct-eGFP-IRES-mCherry-Tubulin expression cassette, MCF-7 cells were co-transfected with pSB-LAGICT and pCMV-SB100X [78], using X-tremeGENE 9 transfection (Roche, Basel, Switzerland) reagent according to the manufacturer’s protocol [77]. Afterwards, the transfected cells were cultured in medium containing G418 (Geneticin) to allow growth of stably transfected cells only. A fluorescence microscope was used to validate the efficiency of the transfection.

### 4.4. Live Cell Imaging by the FLUMIAS Microscope

**Figure 12:**
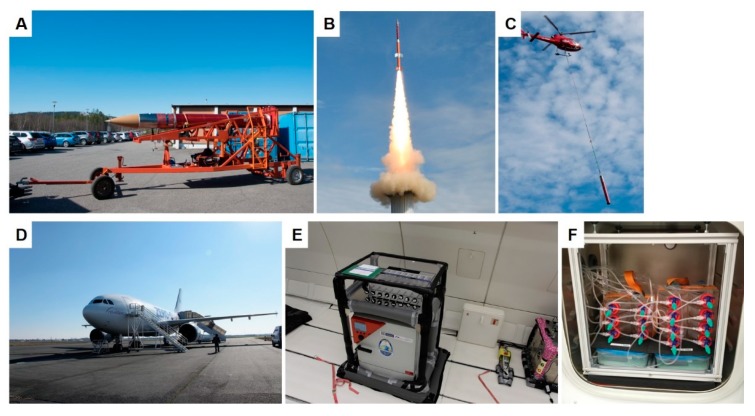
Shows the vehicles and experimental setups of the µ*g*-experiments. (**A**): TX 54 rocket transportation to the launch tower. (**B**): Launch of the TX 54 rocket (courtesy of Airbus, Defense & Space). (**C**): retrieval of the TX 54 rocket with a helicopter. (**D**): Airbus 310 aircraft used for the parabolic flight. (**E**): The incubator used for the experiment. (**F**): Inside of the incubator prior to take off of the flight.

Approximately 7000 MCF-7 cells were seeded into one channel of an ibiTreat μ-slide VI 0.4 (Ibidi, Gräfelfingen, Germany). The slide was temperature controlled and loaded into the FLUMIAS microscope (developed by FEI Munich GmbH [11]) shortly before the launch (Figure 12). Five minutes prior to launch three z-stacks were obtained from pre-selected cells as a ground control. About 75s after launch the microgravity phase was reached, and the microscope started recording the pre-selected cells. Three z-stacks were taken every one minute with 125 ms exposure time. The thickness of the z-stack was 21 μm with 0.5 μm step size. The procedure was repeated four times with a total number of five active phases covering 6 min of microgravity.

After recovery of the image data, a single image was extracted from each z-stack taken during microgravity to allow analysis of all images in the focal plane. The extracted images were deconvolved by Huygens Essential Scientific Volume Imaging software 4.3 and compared to a control image taken on ground.

### 4.5. TEXUS 54 Sounding Rocket Mission

**Figure 13:**
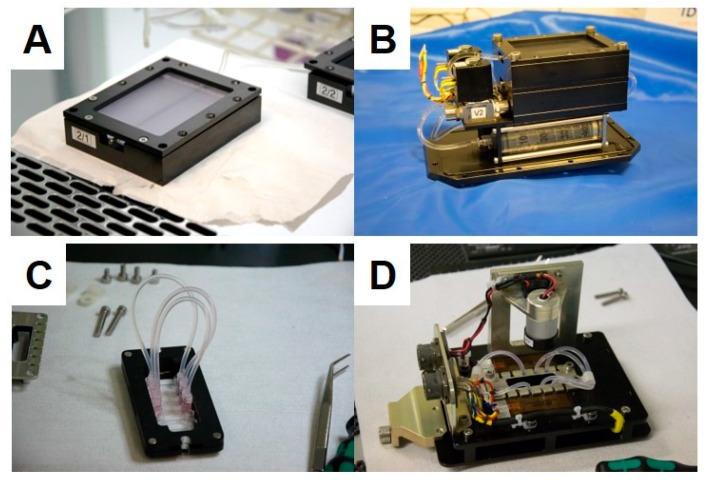
Late access units of the FLUFIX (**A**,**B**) and FLUMIAS (**C**,**D**).

The late access unit (Figure 13) was transferred into the payload, along with the cells shortly before launch. The sounding rocket, used in the TEXUS 54 mission, was composed of the payload and a Brazilian two-stage solid propellant VSB 30 rocket. The rocket was launched on the 13th of May 2018 from ESRANGE space center in Kiruna, Sweden. After launch, the sounding rocket reached an altitude of ~260 km. And it entered microgravity 75 s later after launch. The microgravity phase of < 10^−4^ *g* lasted for ~353 s. Following the microgravity phase, the rocket went back to Earth by a parachute-mediated deceleration. The payload was recovered after landing by a helicopter (Figure 13).

### 4.6. FLUFIX and Immunocytochemistry of MCF-7 Fixed Cells during TEXUS 54

The MCF 7 cells were cultured in 18 well Ibidi slides. Approximately 2500 cells were seeded into each well. 4 slides were cultured on ground at 1*g* to be used as a control. Furthermore, there were four 18 well Ibidi slides on board of the rocket. The cells, on board of the rocket, were fixed with 4% paraformaldehyde (PFA) at two time points. Two Ibidi slides were fixed at the end of the hyper gravity phase and the other two slides were fixed at the end of the microgravity phase. The ground control slides were fixed with 4% PFA in parallel to the launch of the rocket. The slides were recovered and transferred back to the lab in 4% PFA. 

To discard excess PFA, the cells were washed with DPBS three times. Afterwards, the slides were washed with 0.3% Triton X in DPBS for 10 min with agitation which was followed by washing with DPBS. To prevent non-specific binding, the slides were incubated in 3% BSA for one hour. Primary antibodies were added to the slides overnight at 4 °C (Table 1). The following day, the slides were washed with DPBS and the secondary antibody Alex Fluor 488 anti-mouse/anti-rabbit was added for one hour (Table 1). The slides were washed again with DPBS and Alexa Fluor 568 phalloidin was added for one hour, followed by washing with DPBS and mounting with Fluoroshield with DAPI (Sigma). For the slides to be ready for examination, they were incubated overnight at 4 °C. Carl ZEISS LSM 800 Confocal laser scanning microscope was used to examine the cells. Three lasers were used to examine the slides: 488 nm, 561 nm and 405 nm for visualization of Alexa 488, Alexa 568 and DAPI, respectively.

The microscopic images were quantitatively analyzed by ImageJ 1.52b (U.S. National Institutes of Health, Bethesda, MD, USA) to detect the difference in protein expression between different conditions. The intensity of the fluorescence in every photo was measured by ImageJ after the areas of interest were defined by the wand (tracing) tool. Furthermore, all the values were normalized to background fluorescence.

### 4.7. 31st DLR Parabolic Flight Campaign

The parabolic flights were performed from the Bordeaux–Mérignac Airport between 6 and 9 March 2018. The cells were transferred on board of the Airbus 310 to a 37 °C pre-warmed incubator shortly before take-off and they were incubated at 37 °C for the whole time of the flight. The parabolic flight consisted of 31 parabolas. Each parabola had an initial phase of hyper gravity (1.8 *g*) for 22 s during pull up, followed by a microgravity phase for 22 s. At the end of the parabola, there is a second phase of hyper gravity for 22 s during pull out. The flight maneuver is repeated 31 times per flight day [11] (Figure 12).

### 4.8. RNA Isolation and qPCR

During the parabolic flights, the MCF-7 cells were fixed with RNA*later* (Invitrogen by Thermo Fischer Scientific) at the end of the first parabola (P1) and the end of the last parabola (P31). The cells were cultured in T-75 cell culture flasks (75 cm^2^, SARSTEDT) with 10 mL medium in each flask. Each flask had a fixed three-way connector on the lid which was connected with 140 cm tubing to a 50 mL syringe filled with RNA*later*. The RNA*later* was injected manually into the flasks at the designated times. Additional MCF-7 cells were incubated on ground to serve as a ground control. The ground control cells were fixed with RNA*later* in parallel to the samples on board of the flight. After landing, the medium and RNA*later* mixture were thrown away and replaced with 3-5 mL of fresh RNA*later*. Cells were harvested with 25 cm scraper (SARSTEDT) and incubated with RNA*later* at 4 °C in 15 mL tubes until RNA isolation.

All the falcon tubes were centrifuged (2500 g for 10 min at 4 °C), followed by discarding the supernatant. The RNA was isolated afterwards by the RNeasy Mini Kit (Qiagen) according to the manufacturer’s protocol. The quality of the RNA was evaluated with a spectrophotometer. The RNA was converted to cDNA with a High Capacity cDNA reverse Transcription Kit according to the manufacturer’s protocol (Applied Biosystems, Darmstadt, Germany). The the final volume of the reverse transcription reaction mix was 20 µL with 1 µ*g* total RNA added to each reaction mix. The primers were designed using Primer Blast (primer designing tool from NCBI).

A total volume of 13 µL SYBR green reaction mix (Applied Biosystems) was pipetted in each well in a 96 well plate. 1 µL of cDNA was added to each reaction mix with a concentration of 100 µM forward and reverse primers. 7500 Fast Real-Time PCR System (Applied Biosystems) was used to determine the transcription level of targeted genes (Table 2). The program consisted of initial 20 s holding stage of 95 °C followed by cycling stage. The cycling stage consisted of 40 cycles of 3 s at 95 °C and 30 s at 60 °C. A melt curve stage was implemented at the end which consisted of 15 s at 95 °C and 60 s at 60 °C. The data were collected and analyzed by the ΔΔCT method; 18s and TBP were used as reference genes [21].

### 4.9. Western Blot Analysis

Western blot analysis, gel electrophoresis, trans-blotting, and densitometry were carried out following routine protocols as described previously [38,39,40]. Following lysis and centrifugation, aliquots of 30 µ*g* were subjected to SDS-PAGE and Western blotting. The samples attained at the end of the P1 and P31 are compared to 1*g* control samples. Each condition is represented with 4 samples with a total number of 12 samples for all the conditions. The samples were loaded on Criterion XT 4–12% precast gels (Bio-Rad, Hercules, CA, USA) and run for 1h at 150 volts. Proteins were then transferred with a TurboBlot (Bio-Rad) (100 V, 30 min) to a PVDF membrane. Glyceraldehyde -3-phosphate-dehydrogenase (GAPDH) was used as a loading control. Membranes were then blocked for two hours in TBS-T containing 0.3% I Block (Applied Biosystems, Foster City, CA, USA). For detection of the selected antigens (see Table 3), the membranes were incubated overnight at room temperature in TBS-T and 0.3% I Block solutions of the antibodies. Following three washing steps of 5 min, membranes were incubated for additional two hours at room temperature with secondary antibody horseradish peroxidase (HRP)-linked antibody (Cell Signaling Technology Inc., Danvers, MA, USA) diluted 1:4000 in TBS-T and 0.3% I-Block. The respective protein bands were visualized using Bio-Rad Clarity Western ECL (Bio-Rad) and images were captured with Image Quant LAS 4000 mini (GE Healthcare Life Science, Freiburg, Germany). Images of stained membranes were captured on Syngene PXi 4EZ image analysis system (Synoptics, Cambridge, UK) and analyzed using the ImageJ software for densitometric quantification of the respective bands and total protein load [22].

### 4.10. Immunostaining of Fixed MCF-7 Cells Collected during the 31st Parabolic Flight Campaign 

Approximately 20,000 MCF-7 cells were seeded into Ibidi slide flasks two days prior to the flight day. Four flasks were fixed at the end of the first parabola and additional four flasks were fixed at the end of the 31st parabola. Four slide flasks were cultured in parallel on ground to act as a control. Cells were fixed with 4% PFA and transferred to the lab to continue the staining procedure. MMP9, IL 6 and IL-8 were used as primary Antibodies. Alexa Fluor plus 488 goat anti-mouse IgG was used as a secondary antibody. DAPI and phalloidin were used additionally in all the slides (Table 1). The immunostaining procedure was performed as mentioned earlier [22].

### 4.11. Statistical Analysis

GraphPad prism 7.01 (GraphPad Software, Inc., California, USA) was used to analyze the data. The nonparametric Mann–Whitney U test was used as a statistical test of significance. The difference between groups was considered significant when the P-value was less than 0.05.

## 5. Conclusions

The FLUMIAS microscope has now been shown to be an elegant device suitable for live-cell imaging in real microgravity [11,30]. We could show for the first time, significant changes in the F-actin and microtubule cytoskeleton on living MCF-7 breast cancer cells in orbit. MCF-7 cells sense microgravity early and focal adhesion proteins are involved in this process. The actin cytoskeleton is contributing to adhesion and migration of the cancer cells. The early formation of lamellipodia and filopodia is the right step in this direction [66]. Early increases in *VEGFA* and *CXCL8* gene expression as well as the down-regulation of *VCL* mRNA and the reduced E-cadherin protein indicate a change to a more invasive function of the MCF-7 cells exposed to short-term microgravity which supports earlier data with thyroid cancer cells exposed to parabolic flight maneuvers [16]. Our future plan is to investigate these cells for a longer time in space to investigate the behavior of breast cancer cells on the ISS.

## Figures and Tables

**Table 1:** Names, types, classes, companies, and dilutions of all the antibodies that were used for immunostaining.

Antibody Name	Class	Type	Company	Reference no.	Dilution
Matrix Metallopeptidase 9 (MMP9) (2C3)	mouse monoclonal AB	Primary Antibody	Santa Cruz	sc-21733	(1:100)
interleukin 6 (IL6) (E-4)	mouse monoclonal AB	Primary Antibody	Santa Cruz	sc-28343	(1:100)
interleukin 8 (IL8) (C-11)	mouse monoclonal AB	Primary Antibody	Santa Cruz	sc-376750	(1:100)
Vascular Endothelial Growth Factor (VEGF) (c‑term)	Rabbit monoclonal	Primary Antibody	Epitomics, Inc	#1909-1	(1:250)
Alexa fluor plus 488 goat anti-mouse IgG (H + L)	Goat polyclonal	Secondary Antibody	Invitrogen by Thermo Fischer Scientific	A32723	(1:400)
Alexa fluor 488 F(ab’) 2 frangment of goat anti-rabbit igG (H + L)	Goat polyclonal	Secondary Antibody	Invitrogen by Thermo Fischer Scientific	A11070	(1:500)
Alexa Fluor 568 phalloidin	bicyclic peptide toxin	Toxin	Invitrogen by Thermo Fischer Scientific	A12380	one unit in 200 µL per slide
Fluoroshield with DAPI	fluorescent stain	fluorescent stain	Sigma Life science	F6057	no dilution

**Table 2:** List of all the primer sequences used in the quantitative PCR. All the sequences are listed from 5′–3′ direction.

Factor	Primer Name	Sequence 5′–3′
*18S*	18s-F	GGAGCCTGCGGCTTAATTT
	18s-R	CAACTAAGAACGGCCATGCA
*ACTB*	ACTB-F	TGCCGACAGGATGCAGAAG
	ACTB-R	GCCGATCCACACGGAGTACT
*CAV1*	CAV1-F	CCTCCTCACAGTTTTCATCCA
	CAV1-R	TGTAGATGTTGCCCTGTTCC
*CAV2*	CAV2-F	GATCCCCACCGGCTCAAC
	CAV2-R	CACCGGCTCTGCGATCA
*COL1A1*	COL1A1-F	ACGAAGACATCCCACCAATCAC
	COL1A1-R	CGTTGTCGCAGACGCAGAT
*EZR*	EZR-F	GCAATCCAGCCAAATACAACTG
	EZR-R	CCACATAGTGGAGGCCAAAGTAC
*FN1*	FN1-F	TGAGGAGCATGGTTTTAGGAGAA
	FN1-R	TCCTCATTTACATTCGGCGTATAC
*ICAM1*	ICAM1-F	CGGCTGACGTGTGCAGTAAT
	ICAM1-R	CTTCTGAGACCTCTGGCTTCGT
*IL6*	IL6-F	CGGGAACGAAAGAGAAGCTCTA
	IL6-R	GAGCAGCCCCAGGGAGAA
*CXCL8*	IL8-F	TGGCAGCCTTCCTGATTTCT
	IL8-R	GGGTGGAAAGGTTTGGAGTATG
*KRT8*	KRT8-F	GATCTCTGAGATGAACCGGAACA
	KRT8-R	GCTCGGCATCTGCAATGG
*LAMA1*	LAMA1-F	TGACTGACCTGGGTTCAGGA
	LAMA1-R	TGCTAGCACTCCTTGCTTCC
*LAMA3*	LAMA3-F	AAAGCAAGAAGTCAGTCCAGC
	LAMA3-R	TCCCATGAAGACCATCTCGG
*MMP9*	MMP9-F	CCTGGAGACCTGAGAACCAATC
	MMP9-R	TTCGACTCTCCACGCATCTCT
*MSN*	MSN-F	GAAATTTGTCATCAAGCCCATTG
	MSN-R	CCATGCACAAGGCCAAGAT
*TBP*	TBP-F	GTGACCCAGCATCACTGTTTC
	TBP-R	GCAAACCAGAAACCCTTGCG
*TIMP1*	TIMP1-F	GCCATCGCCGCAGATC
	TIMP1-R	GCTATCAGCCACAGCAACAACA
*TLN1*	TLN1-F	GATGGCTATTACTCAGTACAGACAACTGA
	TLN1-R	CATAGTAGACTCCTCATCTCCTTCCA
*TUBB*	TUBB-F	CTGGACCGCATCTCTGTGTACTAC
	TUBB-R	GACCTGAGCGAACAGAGTCCAT
*VEGFA*	VEGFA-F	GCGCTGATAGACATCCATGAAC
	VEGFA-R	CTACCTCCACCATGCCAAGTG
*VCL*	VCL-F	GTCTCGGCTGCTCGTATCTT
	VCL-R	GTCCACCAGCCCTGTCATTT
*PTK2*	FAK1-F	TGTGGGTAAACCAGATCCTGC
	FAK1-R	CTGAAGCTTGACACCCTCGT
*RDX*	RDX-F	GAAAATGCCGAAACCAATCAA
	RDX-R	GTATTGGGCTGAATGGCAAATT
*PAI1*	PAI1-F	AGGCTGACTTCACGAGTCTTTCA
	PAI1-R	CACTCTCGTTCACCTCGATCTTC
*CDH1*	CDH1-F	GCTGGACCGAGAGAGTTTCC
	CDH1-R	CAGCTGTTGCTGTTGTGCTT
*ITGB1*	ITGB1-F	GAAAACAGCGCATATCTGGAAATT
	ITGB1-R	CAGCCAATCAGTGATCCACAA

**Table 3:** List of the names, sources, companies, molecular weight and dilutions of all the antibodies that were used for Western blots.

Antibody Name	Source	Company	Reference no.	MW kDa	Dilution
Anti-Cyclophilin B	Rabbit monoclonal	Abcam	#178397	24	1: 1000
Anti-Cytokeratin	Mouse monoclonal	Sigma	#C1801	68	1: 1000
Anti-E Cadherin	Mouse monoclonal	Abcam	ab1416	97	1:500
Anti-FAK	Rabbit monoclonal	Abcam	ab40794	125	1:1000
Anti-IL-8	Rabbit polyclonal	Abcam	ab7747	11	1:500
Anti-Integrin beta 1	Rabbit monoclonal	Abcam	#134179	88	1: 1000
Anti-Laminin	Rabbit polyclonal	Sigma	#L9393	220	1: 1000
Anti-PAI1	Rabbit polyclonal	Abcam	Ab66705	45	1:1000
Anti-Vinculin	Mouse monoclonal	Abcam	Ab18058	124	1:1000
Anti-β-Actin	Mouse monoclonal	Sigma	A5316	42	1:2000
Beta Tubulin Antibody	Rabbit Polyclonal	Santa Cruz Biotechnology	sc-9104	55	1: 1000
Ezrin	Rabbit polyclonal	Cell Signaling	#3145	81	1:500
Fibronectin	Mouse monoclonal	Invitrogen	#MA5-11981	250	1:1000
GAPDH (14C10)	Rabbit monoclonal	Cell signaling	#5014S	37	1:1000
MMP9	Mouse monoclonal	ThermoFisher	#MA5-14220	92	1: 500
Moesin (Q480)	Rabbit polyclonal	Cell signaling	#3150	78	1:500
Radixin	Rabbit monoclonal	Cell Signaling	#2636S	80	1: 1000
TIMP1	Mouse monoclonal	ThermoFisher	#MA5-13688	28	1: 500

## Abbreviations

2D	two-dimensional
3D	three-dimensional
*ACTB*	Beta-actin
*CAV1*	Caveolin-1
*CAV2*	Caveolin-2
*CDH1*	E-cadherin
*COL1A1*	Collagen type 1 alpha 1
*CXCL8*	Interleukin 8
DAPI	4′,6-diamidino-2-phenylindole
DLR	German Aerospace Center
ECM	Extracellular matrix
ESRANGE	European Space and Sounding Rocket Range
*EZR*	Ezrin
FLUMIAS	spinning-disc Fluorescence Microscopy Analysis System
*FN1*	Fibronectin
FTC-133	Follicular thyroid cancer cell line 133
GFP	Green fluorescent protein
GLOBOCAN	Global Cancer Observatory
IL-6	Interleukin 6
IL-8	Interleukin 8
ISS	International Space Station
ITGB1	Integrin beta 1
*KRT8*	Cytokeratin 8
*LAMA1*	Laminin alpha 1
*LAMA3*	Laminin alpha 3
*LIMA1*	LIM domain and actin-binding protein 1
MCF-7	Michigan cancer foundation
MCS	Multicellular spheroids
*MMP9*	Matrix metalloproteinases 9
*MSN*	Moesin
*MTSS1*	Metastasis suppressor protein 1
P	Parabola
*PAI1*	Plasminogen activator inhibitor 1
PF	Parabolic flight
PFA	Paraformaldehyde
PFC	Parabolic flight campaign
pLAGICT	pcDNA3.1 LifeAct-eGFP-IRES-mCherry-Tubulin
pSB-LAGICT	Sleeping Beauty LifeAct-eGFP-IRES-mCherry-Tubulin
*PTK2*	Protein tyrosine kinase 2; Focal adhesion kinase 1
r-µ*g*	real microgravity
*RDX*	Radixin
RPM	Random positioning machine
s-µ*g*	simulated microgravity
SB	Sleeping beauty
*TIMP1*	Metalloproteinase inhibitor 1
*TLN1*	Talin-1
*TUBB*	Beta tubulin
TX (TEXUS)	‘Technische Experimente unter Schwerelosigkeit’
*VCL*	Vinculin
*VEGFA*	Vascular endothelial growth factor A
WHO	World health organization
